# Room-temperature luminescent lanthanide ionic liquids for optical tagging and anti-counterfeiting

**DOI:** 10.1039/d6ra06049h

**Published:** 2026-09-21

**Authors:** Annie Prasad, Gabriel Hart, Glenieliz C. Menon-Dizon, Krzysztof Pawlak, Chloe Smith, Suzanne Morsch, Anthony J. Fitzpatrick

**Affiliations:** a Dept. of Physical Sciences, School of Science and Technology, Nottingham Trent University Nottingham NG11 8NS UK anthony.fitzpatrick@ntu.ac.uk; b Materials Innovation Factory, University of Liverpool 51 Oxford Street Liverpool L7 3NY UK; c Corrosion and Protection Centre, Department of Materials, The University of Manchester Nancy Rothwell Building, Oxford Rd Manchester M13 9PL UK

## Abstract

The development of robust, scalable materials for next-generation anti-counterfeiting technologies requires the integration of unique optical signatures with practical processability. Here, we report a modular platform of room-temperature luminescent ionic liquids based on trihexyl(tetradecyl)phosphonium salts of lanthanide tris(dipicolinate) complexes (Ln = Eu, Tb, Dy, Sm). These materials combine the sharp, line-like f–f emission characteristics of lanthanides with the inherent advantages of ionic liquids, including negligible vapor pressure, high miscibility, and facile formulation. Crucially, the liquid nature enables direct volumetric mixing of discrete emitters, allowing rapid generation of binary, ternary, and quaternary systems with dense, information-rich emission spectra. The intrinsic emission signatures of each lanthanide are retained in the liquid state, while mixture systems exhibit enhanced spectral complexity without requiring additional synthetic steps. Thermal and calorimetric analyses confirm the preservation of stable room-temperature liquid behaviour, supporting compatibility with established processing and printing technologies. This approach decouples spectral complexity from synthetic complexity, establishing a scalable route to tailorable photonic security materials that offer significant potential for advanced optical tagging, secure identification, and functional soft-material design.

## Abstract 2

The development of robust, scalable materials for next-generation anti-counterfeiting technologies requires the integration of unique optical signatures with practical processability.

## Graphical abstract

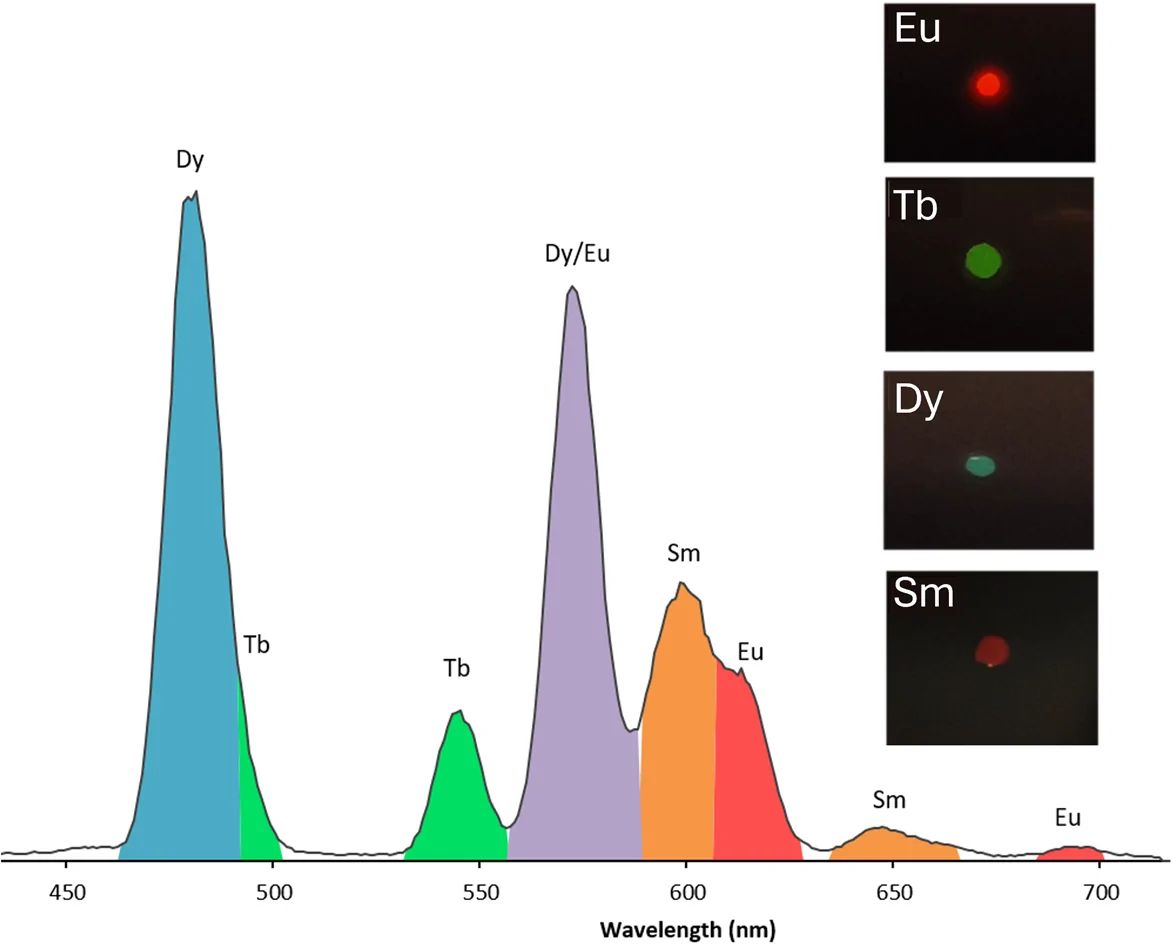

## Introduction

Counterfeiting documents has been a persistent issue throughout history, evolving alongside advancements in technology and posing challenges to security measures. From ancient times, when cowrie shells were counterfeited using materials like ivory and bone, to the Roman Empire's coin clipping and metal casting, counterfeiting techniques have evolved significantly.^1^ Recently, developments in digital printing and graphic arts have revolutionised counterfeiting, making it easier to forge banknotes and important documents.^2^ Consequently, the need for robust security features based on molecular properties that can be quickly verified has become crucial. Many banknotes and documents rely on visible features that can be observed or altered under ultraviolet light illumination. Chemical photoluminescence has played a significant role in exploiting these security features, with substantial advancements made in both fundamental research and practical applications.^3–8^

Despite the progress made, the commercial viability of unique and intriguing materials has been hampered by challenges encountered during the formulation, processing, and fabrication stages. Issues such as solubility, miscibility, robustness, and limitations on printing techniques have hindered their widespread utilisation in industrial settings.^9,10^ To overcome these obstacles, we propose a novel approach that combines two promising chemical properties: lanthanide-based luminescence and ionic liquids.

Lanthanide-based luminescent materials have demonstrated excellent photonic properties and well-defined spectral features, owing to the discrete f–f emission energies.^11–14^ Consequently, it has been extensively utilised across various applications.^15–21^ Emissive lanthanide compounds have been developed in numerous ways by merging structural motifs from materials chemistry, such as metal organic frameworks and nanoparticles, with the luminescent properties. These luminescent materials have been utilised extensively for applications from, sensors for toxic metals,^22^ thermally responsive luminescent materials,^23^ to biomedically useful reagents for imaging guided drug detection and novel imaging contrast agents.^24,25^

Ionic liquids are a class of ionic salts that possess liquid properties below 100 °C and possess remarkable solvation and miscibility characteristics, coupled with negligible vapour pressure.^26–31^ Here we explore the synergistic potential of lanthanide-based luminescence and ionic liquids in enhancing security features against counterfeit materials. Utilising the distinct advantages of these two chemical properties, we aim to address the challenges encountered in formulation, processing, and fabrication, thus paving the way for the wider implementation of advanced security measures in an industrial context. Furthermore, we aim to leverage a new class of mixed lanthanide systems that give rise to more complex spectral features than is currently in use for document security. Luminescent lanthanides can give more identifiable and individual spectra than just standard organic or first row transition metal fluorophores, which tend towards broad emission peaks than can overlap rather than the sharp f–f peaks commonly found in luminescent lanthanide species.^32^ The combination of ease of mixing, formulation, and processing with the increased complexity of the luminescent spectral signature should unlock a brand-new paradigm of security feature to stay ahead of the counterfeiting curve.

There has been previous work pioneering lanthanide and transition metal ionic liquids however the dominant types of complexes consist of metal halide or pseudohalide anions balanced by bulky asymmetric cations.^26,33–38^ This gives a lack of chemical variability or post synthetic modification for tuning the luminescent or liquid properties. Several examples of more intricate metal complex based ionic liquids have been published demonstrating novel properties including magnetic phenomena like spin crossover, green catalysis, and biological extractions.^39–43^ However, most examples or luminescent ionic liquids consist of at best multiple bidentate chelates with limited chemical variation.

## Results and discussion

### Synthesis

The reaction of lanthanide salts (Europium, Terbium, Dysprosium, or Samarium) with deprotonated dipicolinic acid (DPA) results in the formation of the tri anionic Ln(III) complex.^44^ The metathesis of the balancing metal cations with trihexyltetradecylphosphonium (P_66614_) yields strongly luminescent moderately high viscosity lanthanide ionic liquids 1 3(P_66614_)[Eu(DPA)_3_], 2, 3(P_66614_)[Tb(DPA)_3_], and 3, 3(P_66614_)[Dy(DPA)_3_], 4, 3(P_66614_)[Sm(DPA)_3_], Fig. 1.

**Fig. 1:**
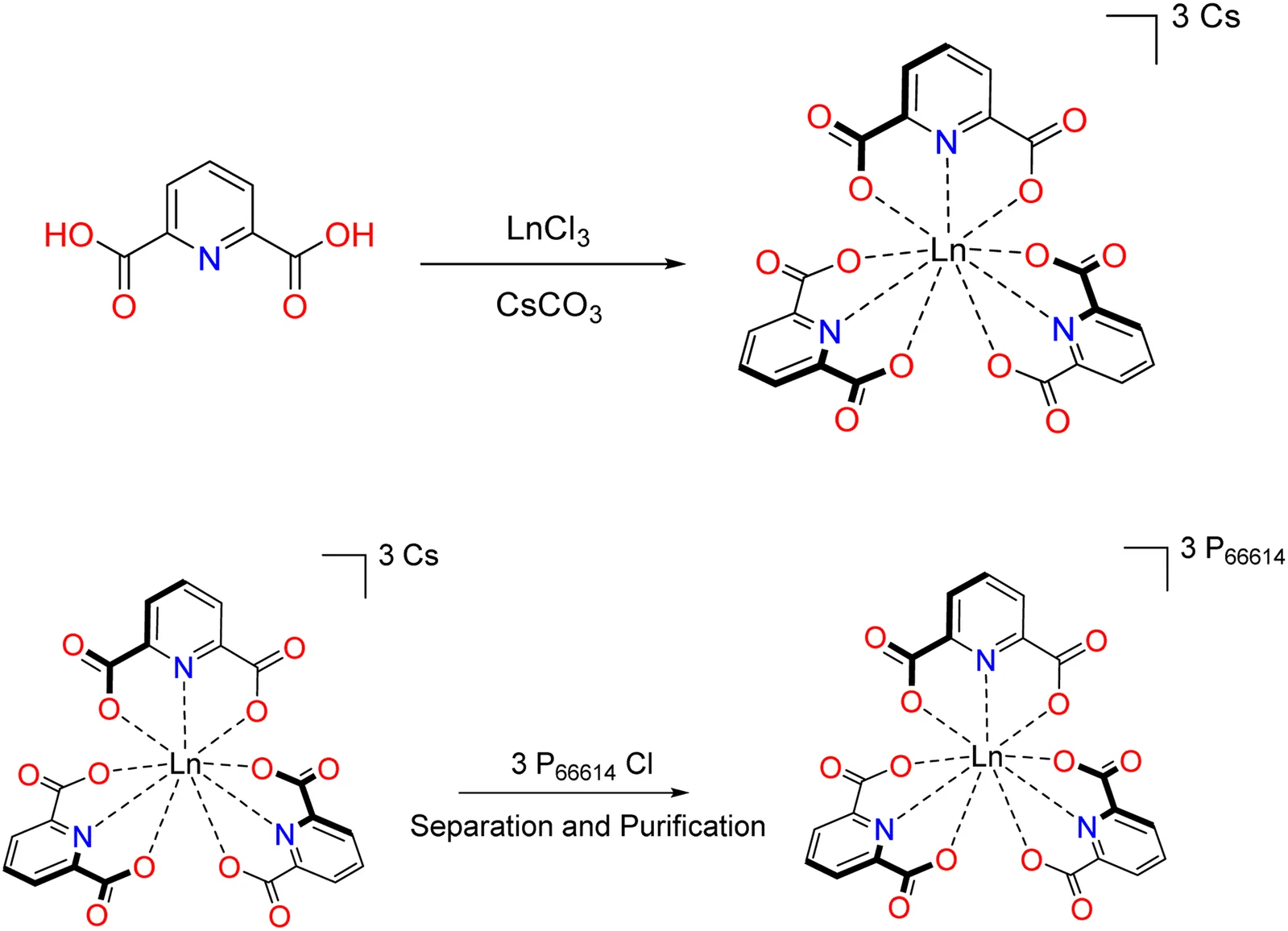
Synthetic route to the luminescent ionic liquids 1, 3(P_66614_)[Eu(DPA)_3_], 2, 3(P_66614_)[Tb(DPA)_3_], 3, 3(P_66614_)[Dy(DPA)_3_], and 4, 3(P_66614_)[Sm(DPA)_3_].

### Physico-chemical properties

While the photochemical properties of each ionic liquid complex are essential to security applications, the need to investigate the physico-chemical properties are equally important. The thermal stability and liquid properties need to be in line with conventional working protocols to be able to sit into the necessary formulation and processing procedures available.

The TGA profiles of the lanthanide ionic liquid complexes 1–4 reflect thermal behaviour characteristic of intrinsically hygroscopic materials, Fig. 2.^45,46^ All four complexes display a small but continuous mass loss below approximately 170 °C, consistent with the gradual release of strongly adsorbed water, an expected feature of ionic liquids, which readily absorb atmospheric moisture. Compared with many lanthanide coordination compounds, which typically exhibit more substantial solvent loss in the 3–8% range, these materials lose relatively little mass in the low temperature region, indicating that only tightly bound water or trace volatile species are present.^47,48^ Beyond 170–180 °C, the mass loss becomes more pronounced, culminating in a sharp decrease between 240–250 °C. This corresponds to the thermal degradation of the dipicolinate complexes due to ligand decarboxylation, producing carbon dioxide as a primary volatile fragment.^49^ Overall, the europium analogue exhibits slightly greater thermal resilience than the Tb, Sm, and Dy complexes, but all display thermal stability comparable to each other.

**Fig. 2:**
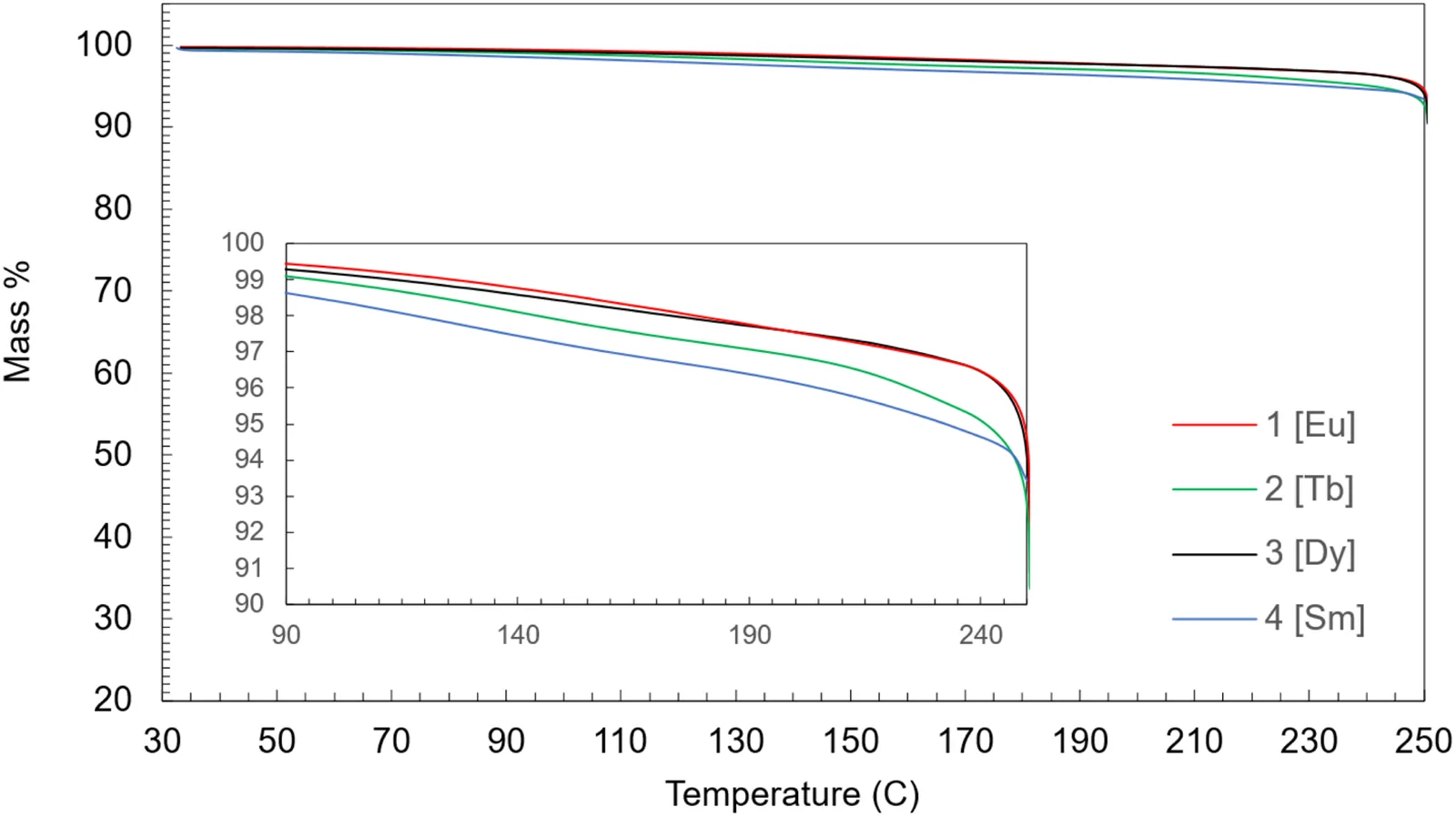
TGA profiles of lanthanide ionic liquid complexes 1–4. All complexes have a minor continuous mass loss below ∼170 °C due to desorption of strongly bound water, followed by a sharper decomposition event at 240–250 °C attributed to dipicolinate decarboxylation.

Differential scanning calorimetry of complex 1 reveals a single, reversible low-temperature transition characteristic of long-chain phosphonium ionic liquids, Fig. 3a.^50,51^ The sample was cooled to −90 °C and allowed to equilibrate, during heating, the sample displays a broad endothermic event reaching a maximum at approximately −78 °C, followed by a gradual return to baseline. Integration of this feature yields an enthalpy change (Δ*H*) of 3.55 kJ mol^−1^, consistent with the low-enthalpy ordering transitions frequently observed in trihexyl(tetradecyl)phosphonium based ILs, where analogous order-disorder or glass-like transitions are commonly reported between −95 and −50 °C and typically exhibit Δ*H* values of only a few kJ mol^−1^.^52–54^ On cooling, post heating, a corresponding exothermic event appears over the same temperature region, confirming the reversibility of the process and indicating a modest hysteresis typical of partial vitrification or ionic-matrix ordering. Above −50 °C, both scans exhibit smooth, featureless baselines with no further thermal transitions up to room temperature, demonstrating that the [Eu(DPA)_3_] does not induce additional phase behaviour within the operational range and that the material remains a stable liquid at ambient conditions. Overall, the DSC profile closely resembles that of related phosphonium ionic liquids, where low-temperature alkyl-chain ordering transitions occur without crystallisation of the ionic network, supporting the classification of the material as a structurally stable room-temperature ionic liquid. Complexes 2, 3, and 4 exhibit almost identical properties, Fig. 3b–d. This therefore supports the retention of the phosphonium cation ionic liquid behaviour with the addition of the luminescence functionality.

**Fig. 3:**
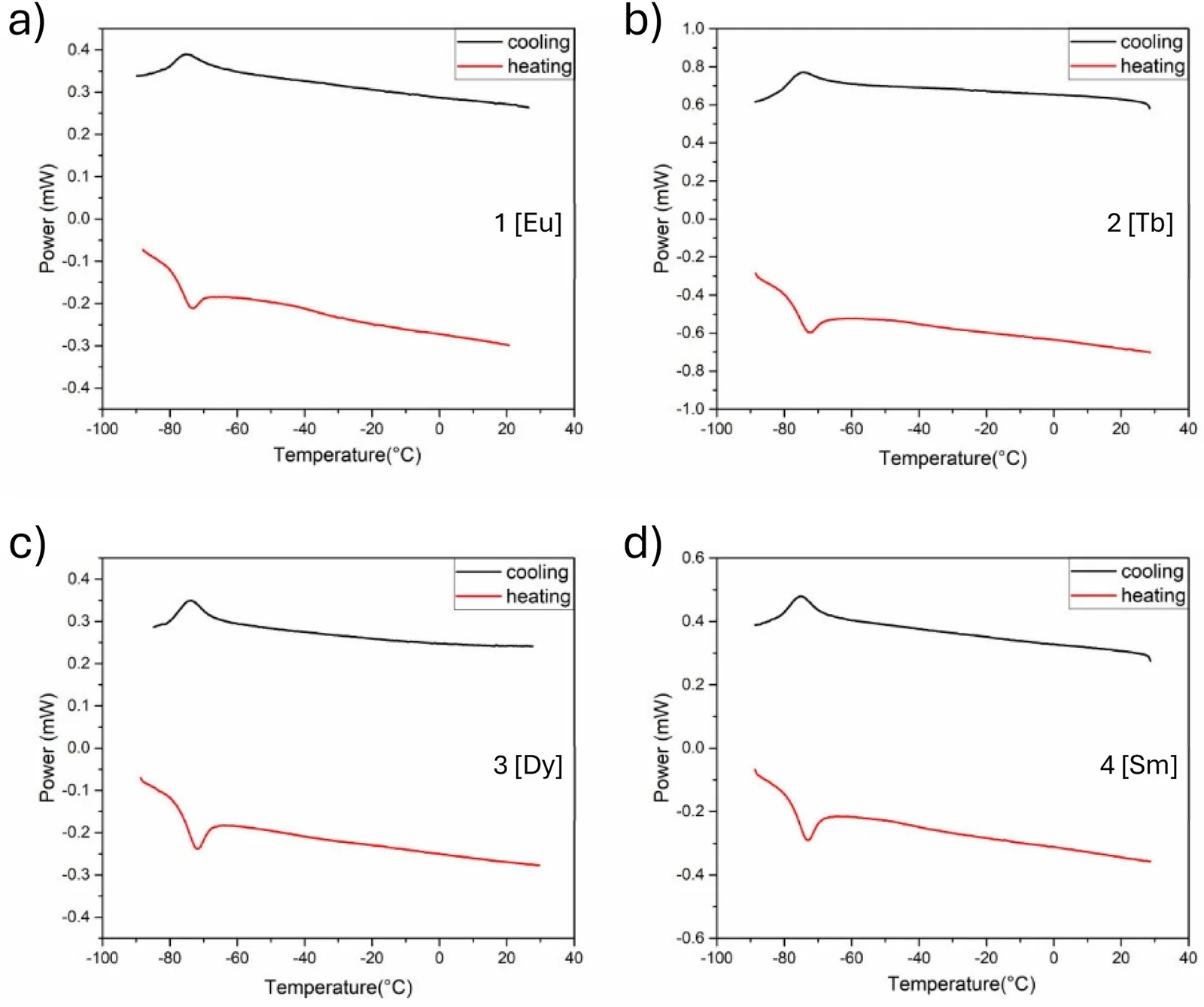
Differential scanning calorimetry: (a) complex 1, cooling (black) and heating (red) scans between −90 and 30 °C. A single reversible thermal event is observed, with an exothermic peak on cooling (onset: −68 °C; maximum: −78 °C) and the corresponding endothermic feature on reheating. Complexes 2, 3, and 4 exhibit almost identical features, (b), (c), and (d), respectively.

### Luminescent properties

Lanthanide luminescence is unique in the fact that the wavelengths observed for a species can be routinely determined from the energies of the f–f energy levels transitions. The main attributing factor to the efficacy of the luminescent system is the sensitiser ligand used to form the complex. The relaxation of energy from the ligand excited singlet state (S^1^) to the triplet state (T^1^) is followed by an energy transfer from T^1^ to the higher energy states in the Ln(III) ion, ^5^D_0_, ^5^D_4_, ^4^F_9/2_, ^4^G_5/2_, for Eu(iii), Tb(iii), Dy(iii), and Sm(iii), respectively, Fig. S.2.^11,55,56^ The photoluminescent properties of each lanthanide containing ionic liquid was first investigated, Fig. 4. All four ionic liquids have strong visibly coloured luminescence when exposed to shortwave UV radiation (insets Fig. 2), with 1, 2, 3, and 4, displaying red, green, aqua, and orange coloured visible emission, respectively. The excitation wavelength used was 315 nm, 302 nm, 321 nm, and 302 nm, for complexes 1, 2, 3, and 4, respectively. This excitation wavelength was determined through excitation/emission spectra, (Fig. S.3). The minor variations in the excitation wavelengths of the arise from a combination of lanthanide contraction and ion-specific electronic structures. While the primary excitation pathway is mediated by the antennae effect of the shared dipicolinic acid (DPA) ligand, the progressive decrease in ionic radius across the lanthanide series alters the local coordination geometry and crystal field strength, subtly shifting the ligand-centred transitions.^57,58^ For complex 1, the peaks at 560 nm, 592 nm, 615 nm, and 695 nm arise from the transitions between the Eu(iii) based ^5^D_0_ to ^7^F_0_, ^7^F_1_, ^7^F_2_, and ^7^F_4_ states, each transition is clearly observable. This agrees with the visible emission colour as the ^5^D_0_ to ^7^F_2_ is the most intense transition centred at 615 nm. Similarly, complex 2, exhibits peaks at 490 nm, 544 nm, 583 nm, 603 nm and 623 nm, representative of the ^5^D_4_ to ^7^F_6_, ^7^F_5_, ^7^F_4_, ^7^F_3_, and ^7^F_2_ transitions associated with Tb(iii) based luminescence. Complex 3 displays peaks at 481 nm, 572 nm, and 638 nm typical of the ^4^F_9/2_ to ^6^H_15/2_, ^6^H_13/2_, and ^6^H_11/2_ transitions associated with Dy(iii) luminescence. Finally complex 4, exhibits peaks at 565 nm, 600 nm, 645 nm, and 710 nm, this correlates with the ^4^G_5/2_ excited state radiatively relaxing to the ^6^H_5/2_, ^6^H_7/2_, ^6^H_9/2_, and ^6^H_11/2_ levels. The successful combination of emissive lanthanides and ionic liquids is evident here as the expected luminescent properties from each of the Ln(III) species have been retained while yielding a room temperature liquid complex.

**Fig. 4:**
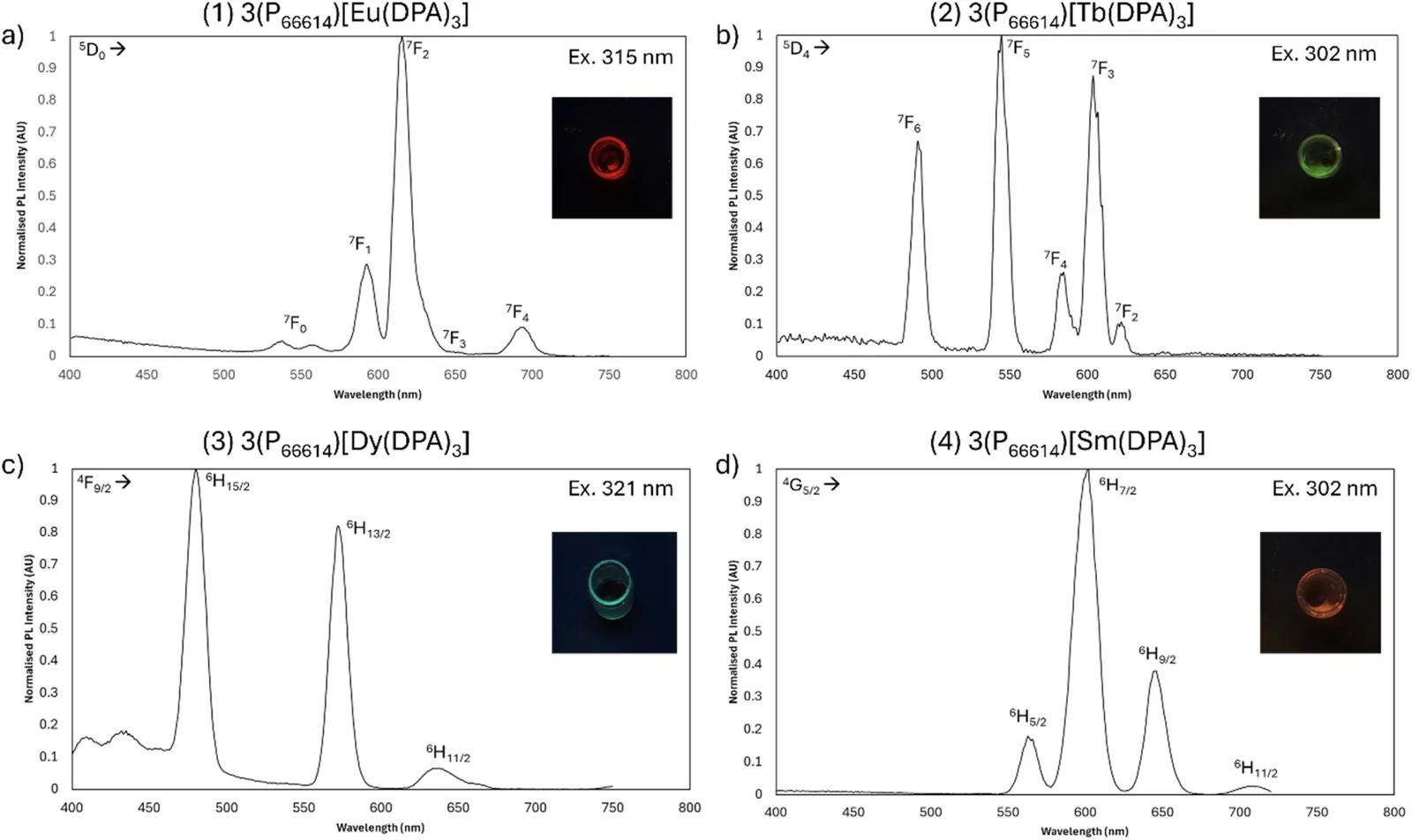
(a) Normalised photoluminescent (NPL) spectrum of complex 1, displaying the Eu(iii) based ^5^D_0_ to ^7^F_0_, ^7^F_1_, ^7^F_2_, and ^7^F_4_ transitions, inset; complex 1 under 254 nm illumination. (b) NPL spectrum of complex 2, displaying the Tb(iii) based ^5^D_4_ to ^7^F_6_, ^7^F_5_, ^7^F_4_, and ^7^F_3_ transitions, inset; complex 2 under 254 nm illumination. (c) NPL spectrum of complex 3, displaying the Dy(iii) based ^4^F_9/2_ to ^6^H_15/2_, and ^6^H_13/2_ transitions, inset; complex 3 under 254 nm illumination. (d) NPL spectrum of complex 4, displaying the Sm(iii) based ^4^G_5/2_ to ^6^H_5/2_, ^6^H_7/2_, ^6^H_9/2_, and ^6^H_11/2_ transitions, inset; complex 4 under 254 nm illumination.

Due to the room temperature liquid nature of ILs 1–4, it is a facile process to synthesise a lanthanide containing ionic liquid with readily known compositions and concentrations of differing lanthanides to yield spectrally complex systems. This removes a layer of formulation complexity found in studies that have used solvent based doping or traditional film synthesis techniques. A known amount can now be measured out and mixed to make a bespoke system. The photoluminescent properties of the binary mixtures were first investigated. Equimolar volumes of each pure parent lanthanide containing ionic liquid were mixed thoroughly and then measured at 254 nm excitation to mimic commercially available high energy UV wavelengths. Using four pure lanthanide based ionic liquids there are six possible unique binary combinations, 5, [EuTb], 6, [EuDy], 7, [EuSm], 8, [TbDy], 9, [TbSm], and 10, [DySm]. The resulting mixes, 5–10, clearly exhibit peaks associated with the parent complex employed. Mixture 5, [EuTb], Fig. 5a, exhibits clear Tb(iii) based luminescent features with peaks at 495 nm, 545 nm, and 650 nm, with Eu(iii) based features at 592 nm, 615 nm, and 695 nm. There is good separation between each peak. This spectrum is far more complex and feature dense that either of the parent systems. Measuring at 254 nm, while commercially applicable, comes with one caveat, the phosphonium cation luminesces at 500 nm. In some cases, this results in peak broadening in the Tb and Dy derived systems due to the overlap of the emitted light between 480–500 nm, as can be observed in mixtures 5 and 6. Where there is no overlapping emissions, the spectra can contain a single extra peak at 500 nm correlating to the cation luminescence (Fig. S.4). This extra peak can complicate the analysis of the spectra to some degree however only when dealing predominately with Tb/Dy based systems that still have unique and identifiable peaks in unaffected areas of the spectrum. Due to similar energy level transitions occurring in some Ln(III) species there are overlap of some peaks when particular combinations are used. Tb(iii) and Eu(iii) exhibits similar peaks around 600–610 nm, 5, Eu(iii) and Sm(iii) exhibit peaks around 600 nm, 7, and Tb(iii) and Sm(iii) exhibit peaks around 540–560 nm, 9. Mixture 10, [DySm], exhibits some unique separation combined with shouldering of the luminescence peaks, however each peak can still be identified and attributed to a particular Ln(III) species.

**Fig. 5:**
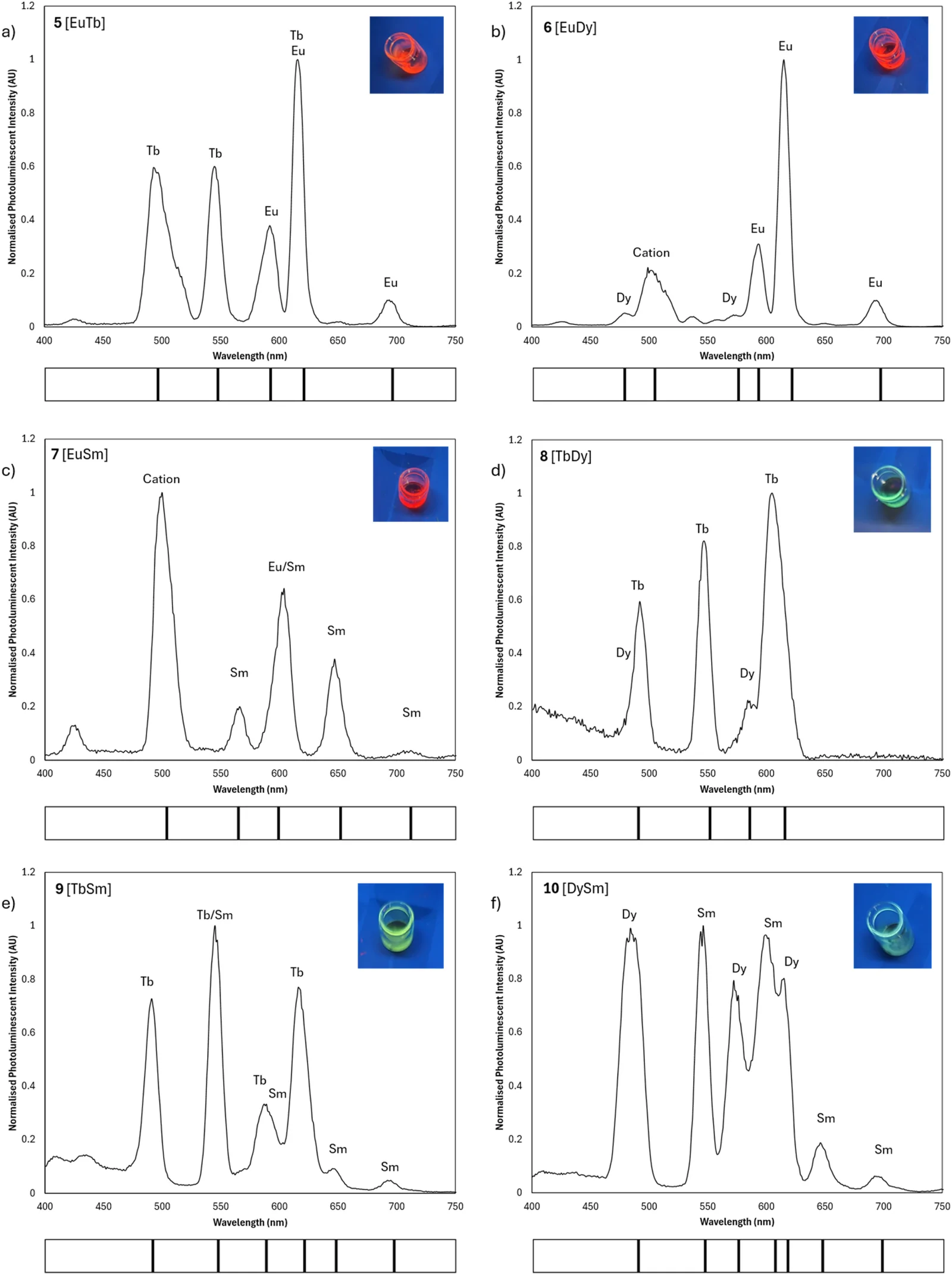
(a) Normalised photoluminescent (NPL) spectrum of binary mixtures of complexes 1–4. (a) NPL spectrum of mixture 5, Eu and Tb mixture, inset; mixture 5 under 254 nm illumination. (b) NPL spectrum of mixture 6, Eu and Dy mixture, inset; mixture 6 under 254 nm illumination. (c) NPL spectrum of mixture 7, Eu and Sm mixture, inset; mixture 7 under 254 nm illumination. (d) NPL spectrum of mixture 8, Tb and Dy mixture, inset; mixture 8 under 254 nm illumination. (e) NPL spectrum of mixture 9, Tb and Sm mixture, inset; mixture 9 under 254 nm illumination. (f) NPL spectrum of mixture 10, Dy and Sm mixture, inset; mixture 10 under 254 nm illumination. Bar under each spectrum corresponds to a generated barcode between 400 nm and 750 nm, marking the wavelength of a major peak in the NPL spectra.

For each binary combination, a unique set of spectral peaks can yield a novel identification and verification paradigm. The wavelength of each spectral peak can be transposed into a linear value, while 400 nm and 750 nm can be taken as arbitrary start and finish values. A rudimentary barcode, that each spectrum can be compared against for future authentication, can then be constructed. Such examples are given under each spectrum in Fig. 5. There is some small shift observed in cases where there is overlap between the f–f transitions of different lanthanide species or the overlap of the cation luminescence and the f–f transitions. This contrasts with many organic fluorophores or transition metal-based complexes that display UV active luminescence, as they produce broad spectral peaks across a wider range of wavelengths.

Moving to the four possible trinary mixtures and single quaternary mixture, mixtures 11–15, we can again see the photoluminescent properties have acted in a synergetic manner and created a dense set of spectral peaks originating from the parent lanthanide ionic liquids used. Several considerations are apparent when appraising the photochemical signatures of the more complex mixtures for use as a security ink. In several cases, there are now more overlapping peaks as were observed in the binary mixtures, there is a large overlap around 615–620 nm when Eu(iii) and Tb(iii) are present together yielding a far more intense peak compared to the rest of the spectrum, as observed in mixture 11 and 12, Fig. 6a and b. It can also be observed that the excitation wavelength can favour one lanthanide over another. Using the commercially relevant 254 nm excitation yields some lower intensity peaks, particularly in the case of Dy(iii) and Sm(iii) containing ionic liquids. This effect is noticeable not only in the spectral data but the visual luminescence of mixtures 11–15 under irradiation is dominated by the colours of Eu(iii) and Tb(iii), red/orange and green, respectively, inset Fig. 6a–e. There is still good separation between the peaks associated to the parent lanthanide emitters, however in the quaternary mixture, 15, we do see a significant overlap between peaks, perhaps proving that four emitters in the visible range give no perceivable improvement to the complexity of the spectrum than any of the trinary mixtures, 11–14.

**Fig. 6:**
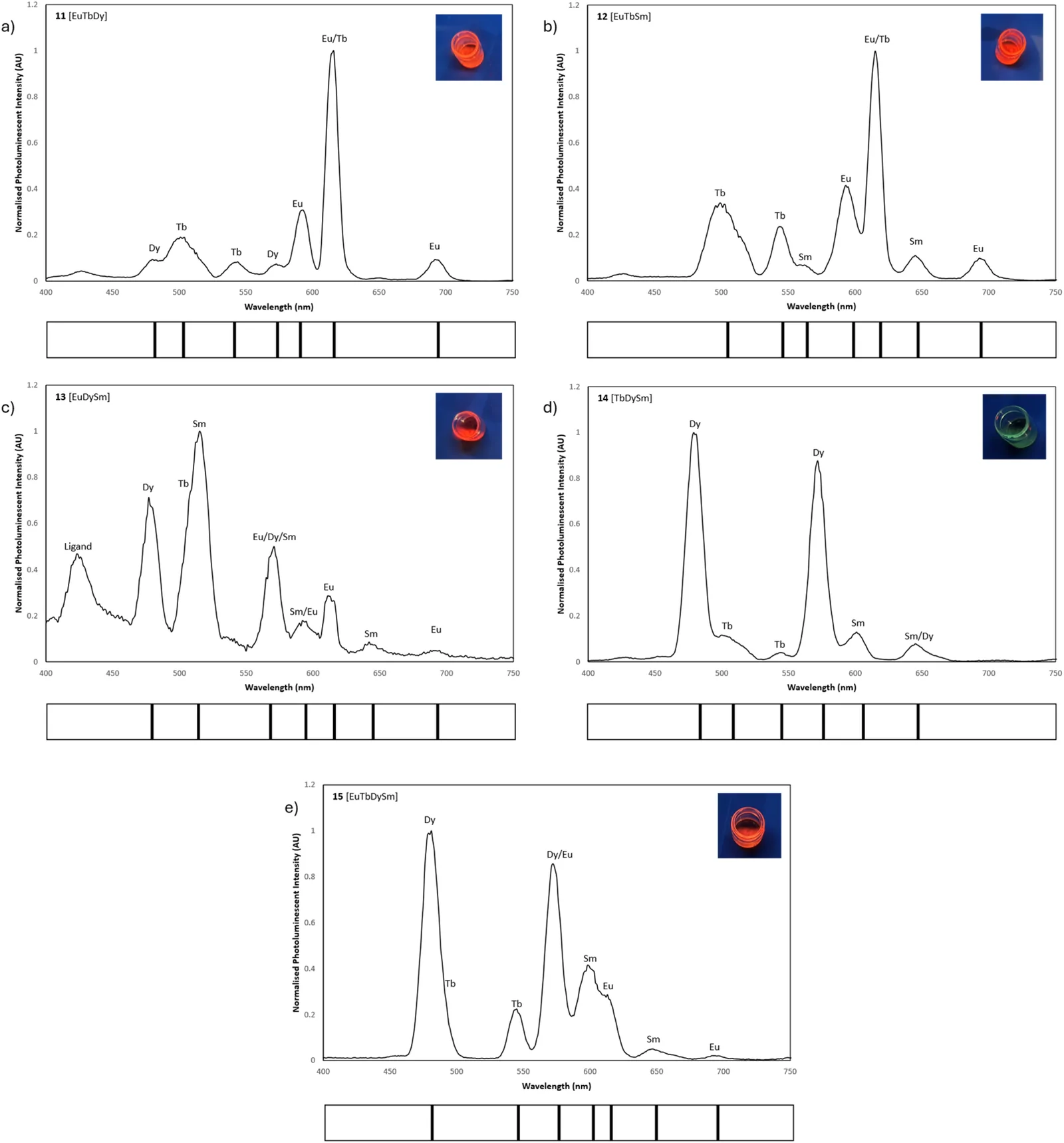
Normalised photoluminescent (NPL) spectrum of binary mixtures of complexes 11–15. (a) NPL spectrum of mixture 11, Eu, Tb, and Dy mixture, inset; mixture 11 under 254 nm illumination. (b) NPL spectrum of mixture 12, Eu, Dy, and Sm mixture, inset; mixture 12 under 254 nm illumination. (c) NPL spectrum of mixture 13, Eu, Tb, and Dy mixture, inset; mixture 13 under 254 nm illumination. (d) NPL spectrum of mixture 14, Tb, Dy and Sm mixture, inset; mixture 14 under 254 nm illumination. (e) NPL spectrum of mixture 15, Eu, Tb, Dy and Sm mixture, inset; mixture 15 under 254 nm illumination.

Time-resolved photoluminescence measurements revealed characteristic monoexponential decay lifetimes of 2.324, 0.254, 0.061 and 0.060 ms for the Eu(iii) (^5^D_0_), Tb(iii) (^5^D_4_), Dy(iii) (^4^F_9/2_) and Sm(iii) (^4^G_5/2_) emissive states, respectively.^59^ Upon incorporation into binary, trinary and quaternary mixtures, systematic perturbations in the excited-state lifetimes were observed, indicative of intermolecular energy migration and cross-relaxation processes.^60–62^ The [EuDy] system exhibited the most pronounced lifetime quenching of the Eu(iii) excited state (*τ* = 1.628 ms), corresponding to an energy-transfer efficiency of 30%, suggesting efficient non-radiative depopulation of the ^5^D_0_ manifold through Eu to Dy sensitisation pathways. Similarly, Sm-containing complexes displayed substantial excited-state coupling, with the [EuDySm] assembly exhibiting the highest calculated transfer efficiency (57% at the Sm(iii) centre), highlighting the role of Sm(iii) as an effective energy acceptor within the lanthanide network. In contrast, Tb(iii) containing complexes generally showed minimal lifetime perturbation, with low transfer efficiencies close to zero or within experimental error, indicating limited electronic communication between the Tb(iii) ^5^D_4_ state and neighbouring lanthanide centres. Notably, despite the increased density of potential donor–acceptor pathways, the quaternary [EuTbDySm] complex exhibited only modest transfer efficiencies (≤7%), suggesting the emergence of competing deactivation channels, energy trapping, or back-transfer processes that limit overall energy migration. Collectively, these results demonstrate that the excited-state dynamics of the mixed-lanthanide dipicolinate framework are highly dependent on metal-ion composition and energy-level matching, with Dy(iii) and particularly Sm(iii) exerting a dominant influence on intramolecular energy-transfer processes and the photophysical relaxation landscape.

The CIE-1931 (ref. 63) colour coordinates were calculated from the emission spectra at 254 nm excitation, Fig. 7. Compounds 1–4, exhibit a chromaticity that correlates with the observed visible emission under 254 nm, namely; red, green, cyan, and orange red, respectively. However, there is a stark contrast in most cases between the calculated chromaticity and the observable colour of emission upon 254 nm excitation for many of the mixtures. Many are in the warm white region with some outliers such as mixture 14 in the cyan region. As stated above, the Eu(iii) containing mixtures predominately exhibit an orange centred visible emission under 254 nm. This could also connect to the low photoluminescent quantum yields (PLQY) for the four parent compounds, 1-Eu, 0.62, 2-Tb, 0.02, 3-Dy, 0.05, 4-Sm, 0.01. Lanthanide tris–dipicolinate complexes can typically display low PLQYs due to inefficient ligand-to-metal energy transfer, competitive ligand fluorescence, and multiple non-radiative decay pathways. The DPA triplet state is often poorly matched to the receiving 4f levels, enabling back-energy transfer or incomplete sensitisation. In addition, the complex is susceptible to vibrational quenching by proximal C–H oscillators, LMCT-mediated deactivation, and structural flexibility that enhances multiphonon relaxation. Together these factors reduce the effective population of the emitting 4f state and suppress overall PLQY.^13,58,64,65^ Compound 1 exhibits an acceptable PLQY and therefore is a possible reason why the orange/red emission is so dominant in the visible emission under 254 nm.

**Fig. 7:**
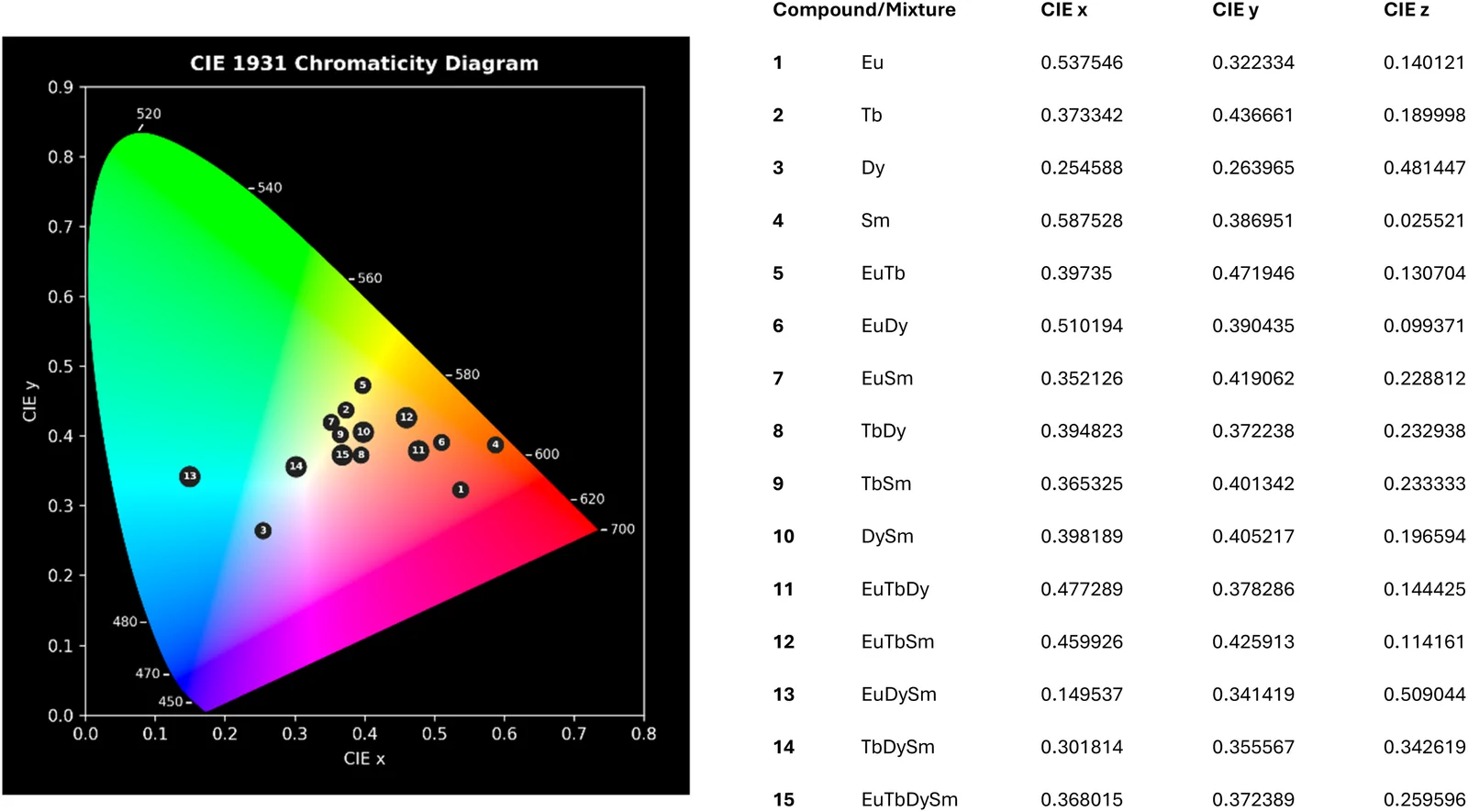
CIE 1931 chromaticity diagram constructed using SpectraChroma v1.0.1 (ref. 66) for 1–15 generated from the luminescence spectra under 254 nm excitation with CIE *x*, *y*, and *z* values tabulated.

To further test the applicability of the luminescent ionic liquids as security dyes, paper was separately dyed with all four single lanthanide liquids, 1–4, and two different combinations; a trinary mixture, 11 [EuTbDy], and a quaternary mixture, 15 [EuTbDySm]. All the papers were imaged under ambient white light, 254 nm, 312 nm and 365 nm, using a Foster + Freeman VSC-40, Fig. 8. From this it can be observed that 254 nm excitation gives the best colour contrast with these systems while allowing similar colours such as in 1 and 15 to appear visually similar but spectroscopically different.

**Fig. 8:**
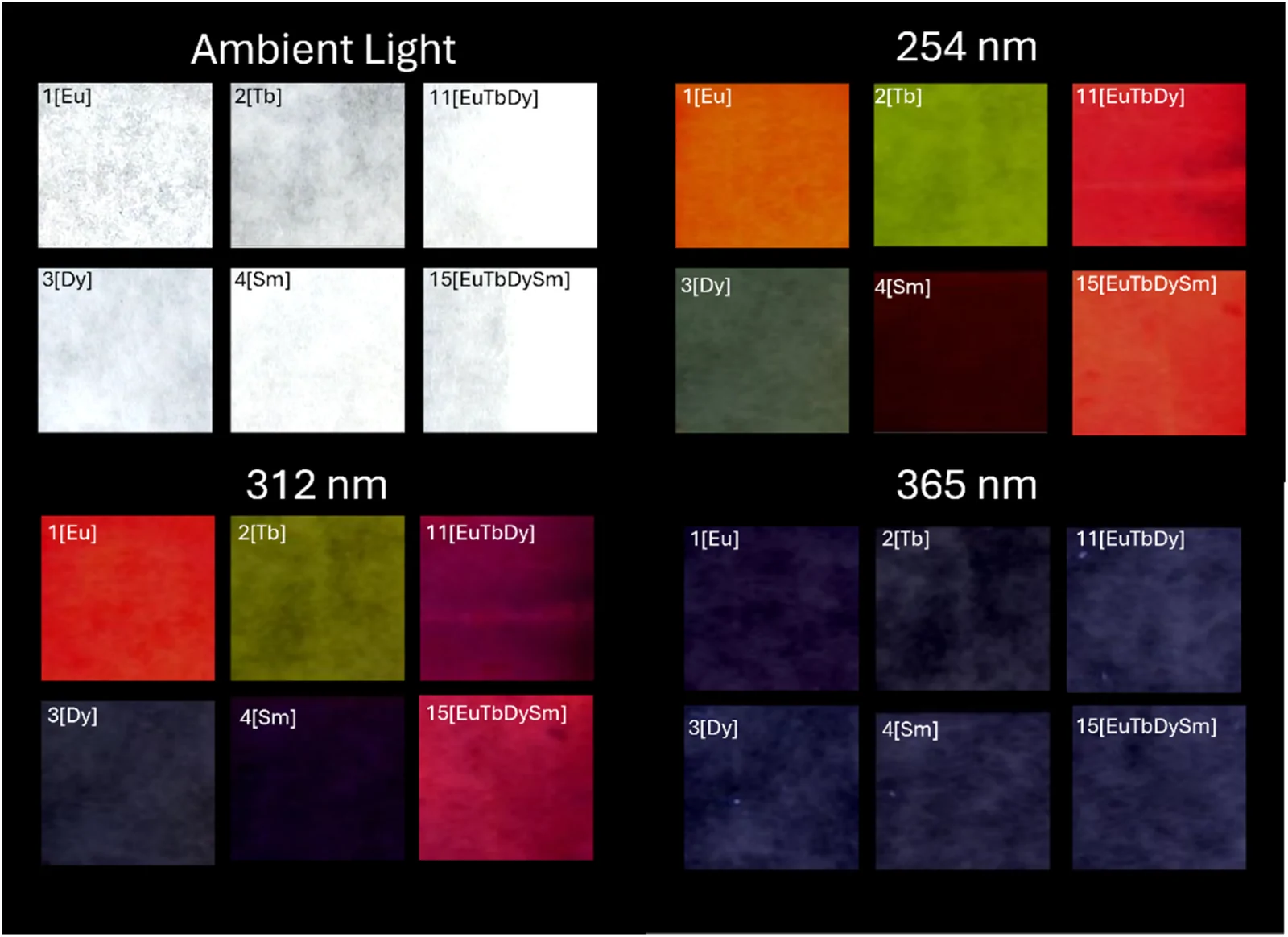
Ionic liquid dyed paper with 1 [Eu], 2 [Tb], 3 [Dy], 4 [Sm], 11 [EuTbDy], and 15 [EuTbDySm]. Illuminated with ambient white light (top left), 254 nm – UVC (top right), 312 nm – UVB (bottom left), 365 nm – UVA (bottom right).

## Conclusion

We report a modular platform of room-temperature luminescent ionic liquids based on trihexyl(tetradecyl)phosphonium salts of [Ln(DPA)_3_], where Ln = Eu, Dy, Tb, or Sm, that couples the narrow-band, line-rich f–f emission of lanthanides with the formulation advantages of ionic liquids. Thermogravimetric analysis indicates limited low-temperature mass loss consistent with tightly bound moisture, followed by principal decomposition attributed to dipicolinate decarboxylation at ∼240–250 °C. Differential scanning calorimetry reveals a single, reversible low-enthalpy ordering event with no crystallisation on reheating, confirming that the phosphonium matrix preserves the liquid state at ambient conditions. Complexes 1–4 retain the characteristic emission of their respective ions while existing as room temperature liquids, enabling solvent-free processing and straightforward volumetric blending. Binary and higher-order mixtures (5–15) yield dense, information-rich spectra composed of resolvable lanthanide transitions; the spectra can be deterministically mapped to barcode-like signatures across 400–750 nm, establishing a simple and scalable route to on-demand optical encoding for anti-counterfeiting. The weak cation-centred emission near 500 nm, while sometimes broadening features under 254 nm excitation, which is an area for further exploration and optimisation. The lifetimes are in line with known lanthanide dipicolinate complexes while the transfer efficiencies remain relatively low between lanthanides when in a mixture. This route is advantageous when looking at the research currently on soft matter lanthanide fluorophores, where sol–gel, film formation and aggregation approaches dominate, leaving the processability issues unaddressed.^67–70^ Together, the data demonstrate that the luminescent functionality is integrated without compromising the intrinsic liquid behaviour required for practical ink formulation. We also demonstrate a proof-of-concept paper dyeing by simple addition of the ionic liquid to a paper substrate followed by UV imaging. By decoupling spectral complexity from synthetic complexity mixing known volumes of stable, neat liquids to encode unique optical fingerprints; this chemistry offers a manufacturable pathway to robust security features compatible with established printing workflows. Future work will target excitation-wavelength optimisation to balance emitter contributions (particularly Dy and Sm under 254 nm), expansion of the ligand/cation library to tune photophysics and viscosity, quantitative lifetime-based read-out, and durability testing under realistic processing and ageing conditions. We anticipate that this modular approach will help maintain a technological lead against document counterfeiting while opening broader opportunities for optical tagging and secure identification.

## Author contributions

A. P. – methodology, investigation, formal analysis, data curation, writing – original draft, writing – review & editing, visualization, conceptualization. G. H. – methodology, investigation, data curation. G. C. M- D. – methodology, investigation. K. P. – investigation, methodology. C. S. – investigation, methodology, S. M. – supervision, A. J. F. – funding acquisition, supervision, writing – original draft, writing – review & editing, conceptualization, project administration, visualization.

## Conflicts of interest

The authors declare no conflict of interest.

## Supplementary Material

RA-016-D6RA06049H-s001

## Data Availability

The data supporting this article have been included as part of the supplementary information (SI) any further information or data is available from the corresponding author upon reasonable request. Supplementary information is available. See DOI: https://doi.org/10.1039/d6ra06049h.
