# Supplementary material for: Room-temperature luminescent lanthanide ionic liquids for optical tagging and anti-counterfeiting
Source: RSC Adv. 2026 Sep 21;16(44):56155–65. doi: 10.1039/d6ra06049h (PMC13592506; doi:10.1039/d6ra06049h)
Supplement: Supplementary file 1 [file RA-016-D6RA06049H-s001.pdf]

Supplementary Information (SI) for RSC Advances.
This journal is © The Royal Society of Chemistry 2026

Room Temperature Luminescent Lanthanide Ionic Liquids for Optical Tagging and Anti-Counterfeiting

Annie Prasad,[a] Gabriel Hart,[a] Glenieliz C. Menon-Dizon,[a] Krzysztof Pawlak,[b] Chloe Smith,[c] Suzanne Morsch,[c] Anthony J. Fitzpatrick*[a]

a Dept. of Physical Sciences, School of Science and Technology, Nottingham Trent University, Nottingham NG11 8NS, UK

b Materials Innovation Factory, University of Liverpool, 51 Oxford Street, Liverpool L7 3NY, UK

c Corrosion and Protection Centre, Department of Materials, The University of Manchester, Nancy Rothwell Building, Oxford Rd, Manchester M13 9PL, UK

1. Instrumentation
2. Synthesis
3. UV Vis
4. PL - Luminescence measurements
5. Lifetime Studies
6. FTIR

1. Instrumentation

Emission spectroscopy of the compounds was done using a Varian Cary Eclipse Fluorescence Spectrometer. The cuvettes used are quartz, 1.5 mm X 1.5 mm pathlength. The software used is CaryScan. Agilent Technologies Cary 630 FTIR was used to measure IR spectra and Agilent Technologies Cary 8454 UV Vis spectrometer for obtaining UV Vis spectra. A Mettler Toledo TGA/DSC 3 + STAR System is for thermogravimetric analysis. Software used is STARe. ViscoQC viscometer was used for viscosity readings, on loan from Anton Parr. Preliminary emission experiments were done on Infinite M200PRO TECAN spectrometer using a Hellma quartz 96-well plate.

2. Synthesis

Synthesis of 1, 3(P_{66614})[Eu(DPA)$_3$]

3Cs[Eu(DPA)] was synthesised following literature protocols[1] where europium(III) chloride, dipicolinic acid, and cesium carbonate were mixed in water and refluxed for 3 hours before precipitation and washing with water to yield a polycrystalline powder. 0.780 g of 3Cs[Eu(DPA)] was then added to 30 ml of deionised water, while 0.515 g of trihexyl(tetradecyl)phosphonium chloride was added to 30 ml of dichloromethane. Both solutions were stirred for 20 min before mixing, and a further 30 min stirring before separation of the organic layer and removal of the solvent yielded a viscous colourless liquid. IR (ATR, cm^{-1}): 2923, 2847, 1625, 1461, 1379, 1095, 723, UV (λ_{Max}): 203,262,270, 279 , % Yield: 21%.

Synthesis of 2, $3(P_{66614})[Tb(DPA)_3]$

3Cs[Tb(DPA)] was synthesised using the same protocol and yielded a polycrystalline white powder. 0.350 g of 3Cs[Tb(DPA)] was dissolved in 30 ml of deionised water, while 0.515 g of trihexyl(tetradecyl)phosphonium chloride was dissolved in 30 ml of dichloromethane. Both solutions were stirred for 20 minutes before mixing and stirred for 1 hour before separation of the organic layer, followed by removal of the solvent, yielding a colourless liquid. IR(ATR, cm^{-1}): 2924, 2847, 1625, 1462, 1377, 723. UV (λ_{Max}): 203,262,270, 279. % Yield: 23%.

Synthesis of 3, $3(P_{66614})[Dy(DPA)_3]$

3Cs[Dy(DPA)] was synthesised using the same protocol and yielded a white poly-crystalline powder. 0.350 g of 3Cs[Dy(DPA)] was dissolved in 30 ml of deionised water, and 0.519 g of trihexyl(tetradecyl)phosphonium chloride was dissolved in 30 ml of dichloromethane. Both solutions were stirred for 20 minutes before mixing and stirred for 1 hour at room temperature before separation of the organic layer, followed by removal of the solvent, yielding colourless liquids. IR (ATR, cm^{-1}): UV(λ_{Max}) : 200,262,270,279 nm. % Yield: 28%

Synthesis of 4, $3(P_{66614})[Sm(DPA)_3]$

3Cs[Dy(DPA)] was synthesised using the same protocol and yielded a white poly-crystalline powder. 0.350 g of 3Cs[Dy(DPA)] was dissolved in 30 ml of deionised water, and 0.519 g of trihexyl(tetradecyl)phosphonium chloride was dissolved in 30 ml of dichloromethane. Both solutions were stirred for 20 minutes before mixing and stirred for 1 hour in room temperature before separation of the organic layer, followed by removal of the solvent, yielding colourless liquids. IR (ATR, cm^{-1}): UV(λ_{Max}) : 202,262,270,279 nm. % Yield: 25%.

3. UV Vis spectroscopy

UV(λ_{Max}) = 202 nm for all ionic liquids.

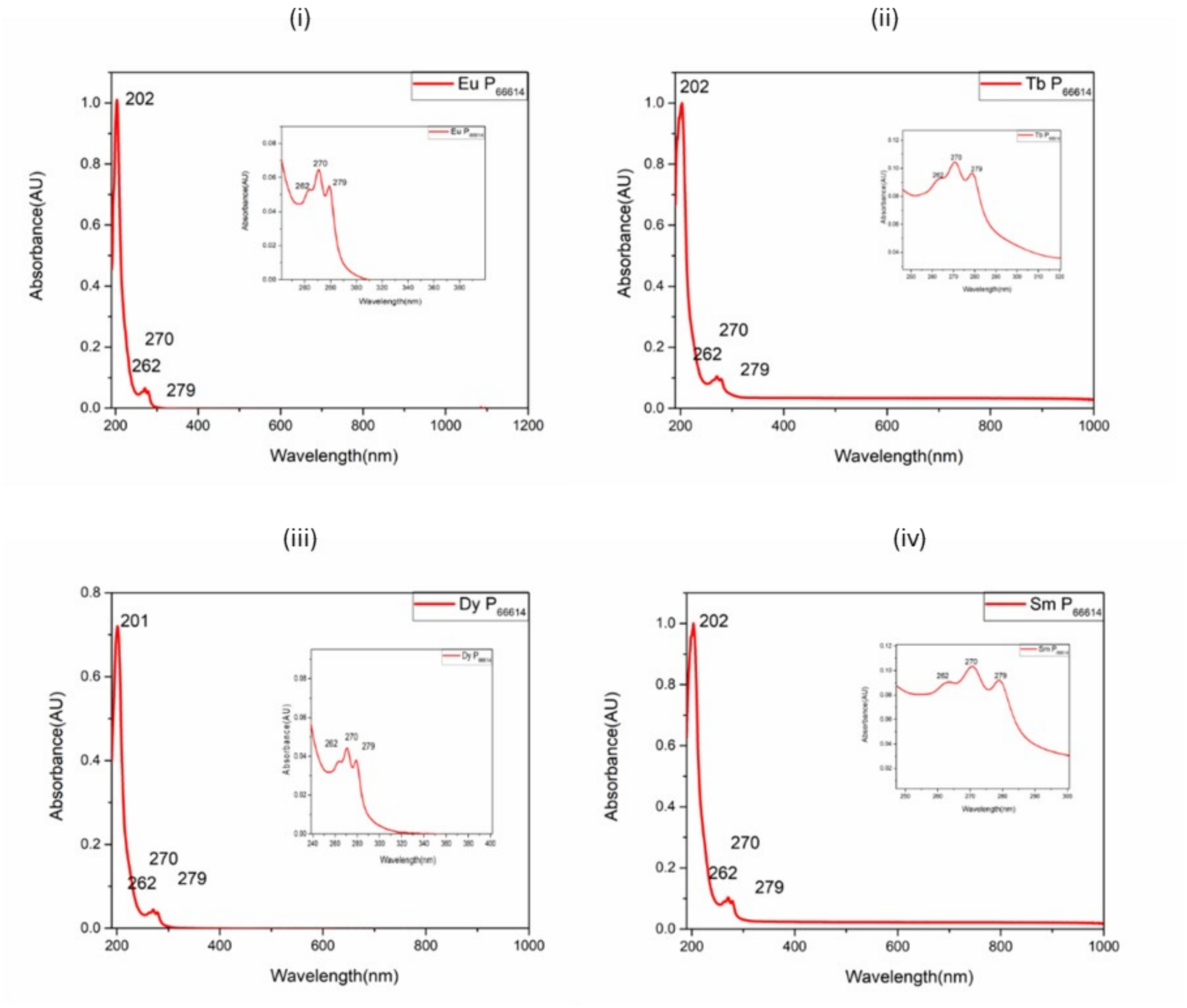

Figure S.1: UV Vis spectra of all 4 ionic liquids. Solvent used is ACN. The cuvette used was a quartz 3x3 mm pathlength.

4. PL measurements

Emission spectroscopy of the compounds was done using a Varian Cary Eclipse Fluorescence Spectrometer. The cuvettes used are 1.5 mm X 1.5 mm pathlength. The software used is CaryScan. A synchronous scan with slit width 10 and delta 300 was measured for each pure ionic liquid, and the Excitation maxima is used to excite to obtain emission spectra of each compound. The Jablonski diagram was derived from the excitation/emission spectra.

Relaxation Pathways

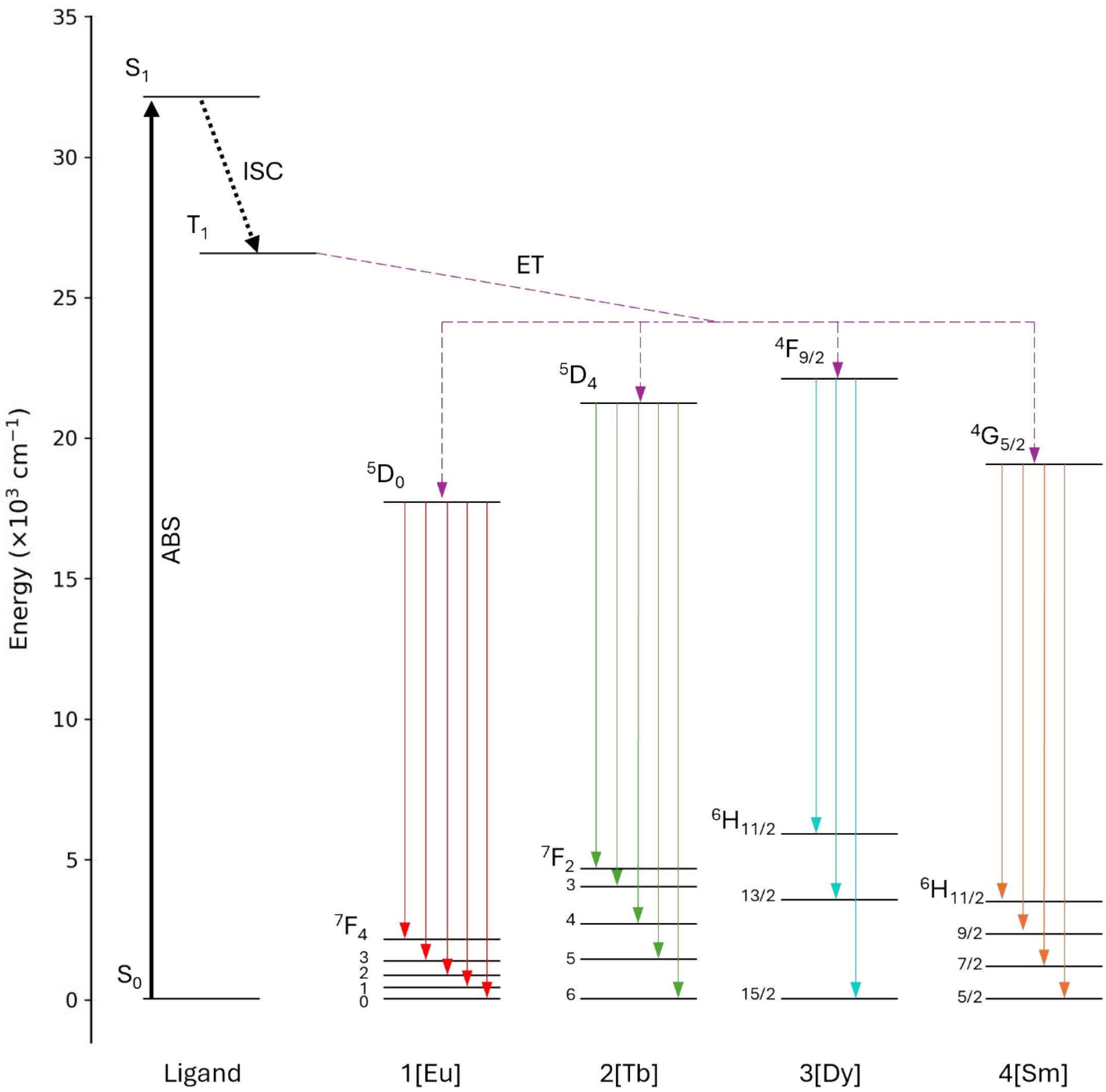

Figure S.2: Jablonski Diagram derived for the single lanthanide ionic liquid.

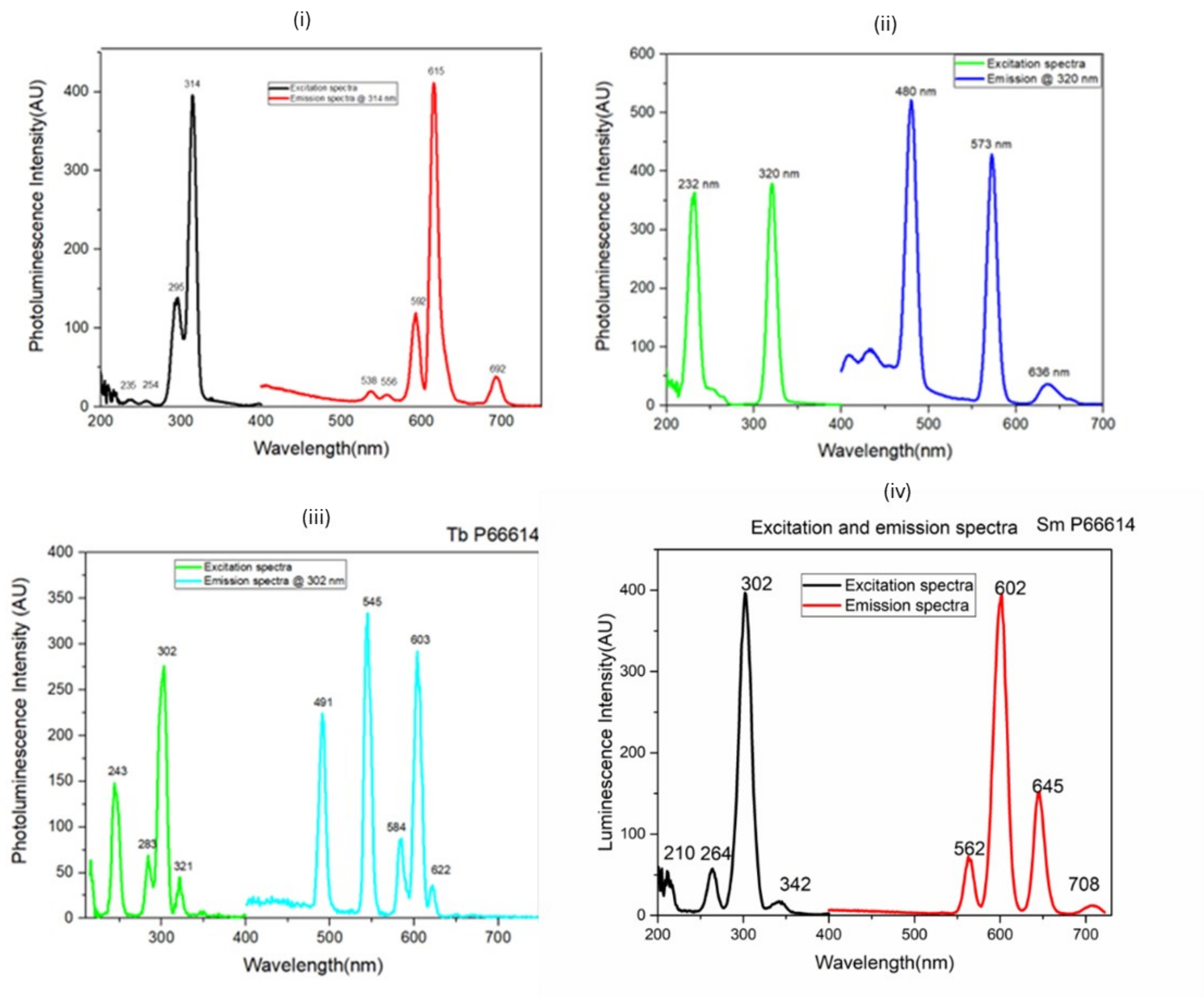

Figure S.3: Excitation/Emission Spectra for 1,2,3, and 4.

Emission spectra of P_{66614} ionic liquids at 365 nm are as follows.

Cation fluorescence can impact the emission bands. The emission spectra of trihexyltetradecyl phosphonium chloride at 254 nm, 365 nm and synchronous scan for excitation and emission spectra is as follows.

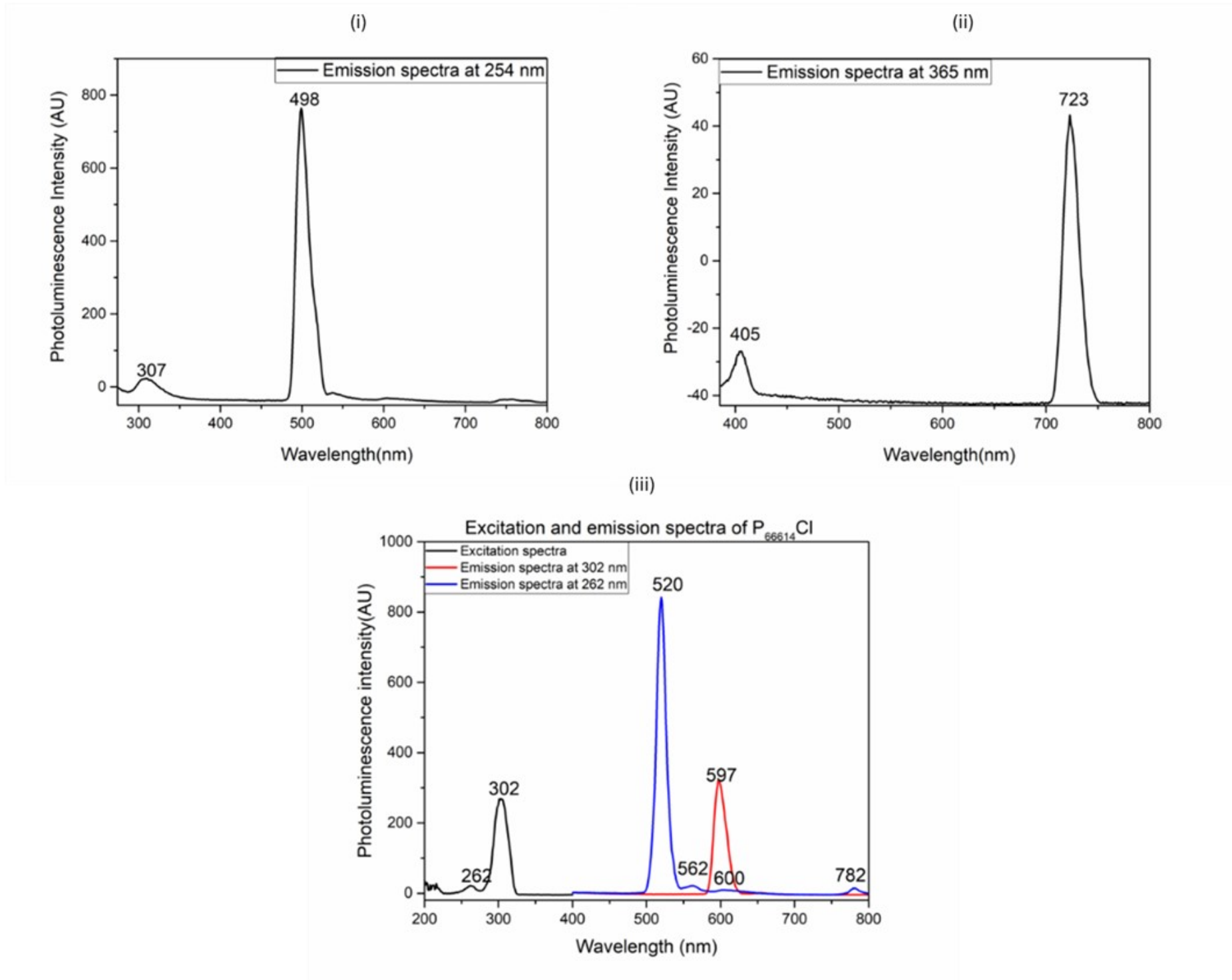

Figure S.4: Excitation/Emission Spectra for P_{66614} Cl

5. Lifetime Measurements.

The lifetime was determined from fitting the monoexponential decay parameters for the pure single lanthanide ionic liquid at the excitations stated previously.

The mixtures were then monitored at the indicative peaks for each lanthanide with the corresponding excitation. 615, 545, 480, and 602 nm for Eu, Tb, Dy, and Sm, respectively.

Table S.1: Photoluminescent Lifetimes of (ms) of **1-15**

		τ (ms)			
	Lanthanide	Eu	Tb	Dy	Sm
1	Eu	2.324			
2	Tb		0.254		
3	Dy			0.061	
4	Sm				0.06
5	EuTb	2.389	0.296		
6	EuDy	1.628		0.0516	
7	EuSm	2.257			0.0403
8	TbSm		0.285		0.0605
9	DySm			0.0573	0.041
10	DyTb		0.257	0.0583	
11	EuTbDy	2.152	0.237	0.057	
12	EuTbSm	2.267	0.286		0.048
13	EuDySm	2.044		0.055	0.026
14	DyTbSm		0.273	0.056	0.055
15	EuTbDySm	2.343	0.271	0.0566	0.0565

The energy transfer efficiencies were calculated based on experimentally derived lifetimes for the single lanthanide ionic liquids compared to the lifetime of the other lanthanide present as acceptors suing the following equation. [2]

$$E = 1 - \left(\frac{\tau_{\mathrm{DA}}}{\tau_{\mathrm{D}}} \right)$$

Where E is the energy efficiency, τ_{DA} is the lifetime of the donor in the presence of an acceptor and τ_D is the lifetime of the donor only.

Table S.2: Energy transfer efficiencies for **5-15**

		Energy Transfer Efficiency			
	Lanthanide	Eu	Tb	Dy	Sm
5	EuTb	-3%	17%		
6	EuDy	30%		15%	
7	EuSm	3%			33%
8	TbSm		12%		-1%
9	DySm			6%	32%
10	TbDy		-1%	4%	
11	EuTbDy	7%	7%	7%	
12	EuTbSm	2%	13%		20%
13	EuDySm	12%		10%	57%
14	TbDySm		-7%	8%	8%
15	EuTbDySm	-1%	-7%	7%	6%

7. FTIR

Agilent Technologies Cary 630 FTIR was used to measure IR spectra. FTIR spectra of P66614 Chloride, dipicolinic acid, and **1**, **2**, **3**, and **4**.

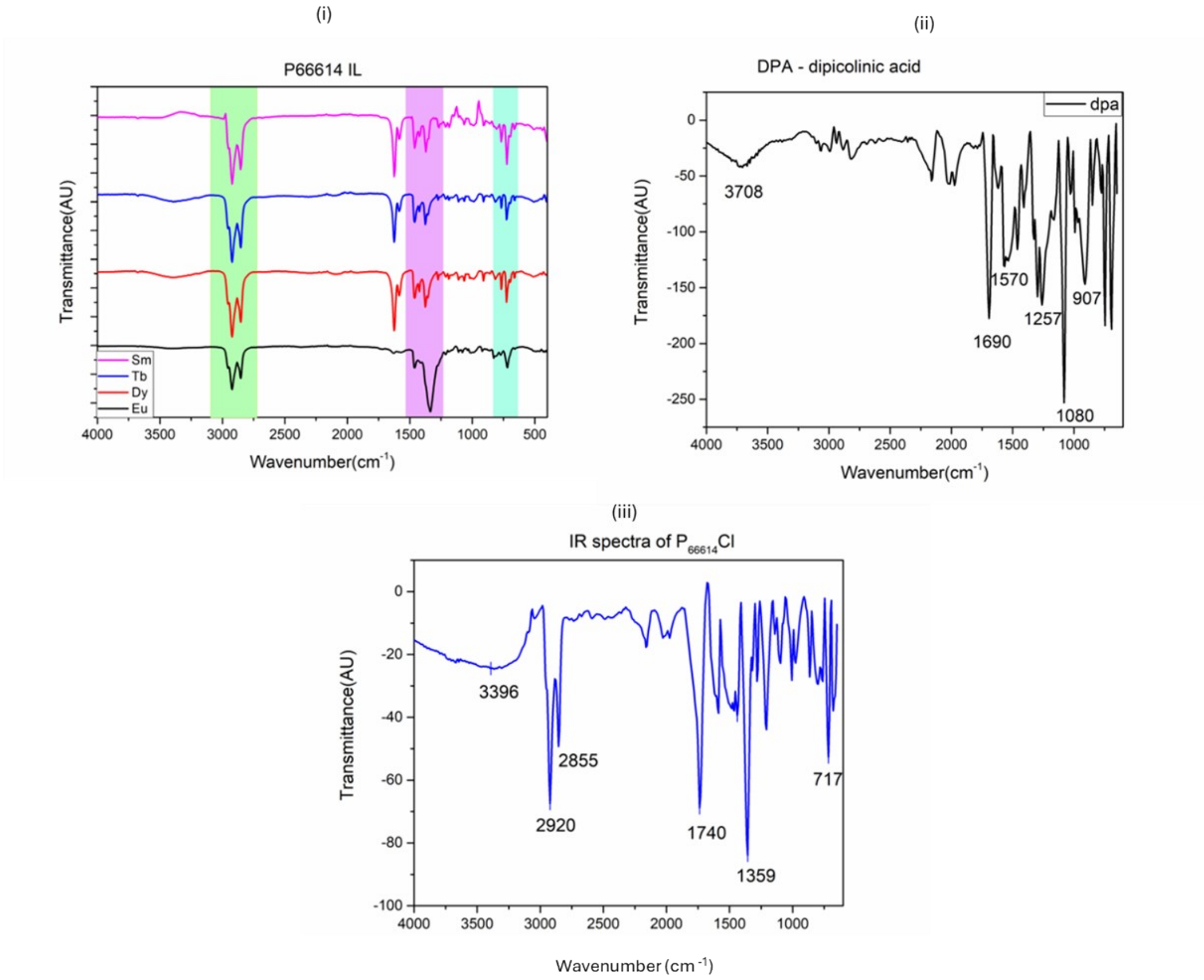

Figure S.5: FTR-IR spectra of (i), **1**-**4**, (ii) dipicolinic acid, and (iii) P_{66614} Cl

1 P. A. Brayshaw, J. C. Bünzli, P. Froidevaux, J. M. Harrowfield, Y. Kim and A. N. Sobolev, *Inorg. Chem.*, 1995, **34**, 2068–2076.

2 T. Boltersdorf, F. N. E. Gavins and N. J. Long, *Chem. Sci.*, 2021, **12**, 8740–8745.
